# Correlation between abnormal brain network activity and electroencephalogram microstates on exposure to smoking-related cues

**DOI:** 10.1192/bjo.2022.641

**Published:** 2023-01-31

**Authors:** Hefan Gan, Junjie Bu, Ginger Qinghong Zeng, Huixing Gou, Mengyuan Liu, Guanbao Cui, Xiaochu Zhang

**Affiliations:** Department of Radiology, the First Affiliated Hospital of USTC, Hefei National Research Center for Physical Sciences at the Microscale and School of Life Science, Division of Life Science and Medicine, University of Science and Technology of China, Hefei, China; School of Biomedical Engineering, Anhui Medical University, Hefei, China; Institute of Advanced Technology, University of Science and Technology of China, Hefei, China; Department of Psychology, School of Humanities and Social Science, University of Science and Technology of China, Hefei, China; Application Technology Center of Physical Therapy to Brain Disorders, Institute of Advanced Technology, University of Science and Technology of China, Hefei, China; Department of Radiology, the First Affiliated Hospital of USTC, Hefei National Research Center for Physical Sciences at the Microscale and School of Life Science, Division of Life Science and Medicine, University of Science and Technology of China, Hefei, China; Department of Psychology, School of Humanities and Social Science, University of Science and Technology of China, Hefei, China; Application Technology Center of Physical Therapy to Brain Disorders, Institute of Advanced Technology, University of Science and Technology of China, Hefei, China; Biomedical Sciences and Health Laboratory of Anhui Province, University of Science and Technology of China, Hefei, China.

**Keywords:** Nicotine-dependent smokers, smoking cue reactivity, imagery scripts, cue-induced craving, EEG microstate analysis

## Abstract

**Background:**

Research into neural mechanisms underlying cue-induced cigarette craving has attracted considerable attention for its significant role in treatments. However, there is little understanding about the effects of exposure to smoking-related cues on electroencephalogram (EEG) microstates of smokers, which can reflect abnormal brain network activity in several psychiatric disorders.

**Aims:**

To explore whether abnormal brain network activity in smokers on exposure to smoking-related cues would be captured by EEG microstates.

**Method:**

Forty smokers were exposed to smoking and neutral imagery conditions (cues) during EEG recording. Behavioural data and parameters for microstate topographies associated with the auditory (A), visual (B), salience and memory (C) and dorsal attention networks (D) were compared between conditions. Correlations between microstate parameters and cigarette craving as well as nicotine addiction characteristics were also analysed.

**Results:**

The smoking condition elicited a significant increase in the duration of microstate classes B and C and in the duration and contribution of class D compared with the neutral condition. A significant positive correlation between the increased duration of class C (smoking minus neutral) and increased craving ratings was observed, which was fully mediated by increased posterior alpha power. The increased duration and contribution of class D were both positively correlated with years of smoking.

**Conclusions:**

Our results indicate that smokers showed abnormal EEG microstates when exposed to smoking-related cues compared with neutral cues. Importantly, microstate class C (duration) might be a biomarker of cue-induced cigarette craving, and class D (duration and contribution) might reflect the relationship between cue-elicited activation of the dorsal attention network and years of smoking.

As a psychiatric disorder, nicotine addiction is one of the most serious public health problems, causing more than 8 million deaths each year around the world.^1^ A core feature of nicotine addiction is smoking cue reactivity, which refers to the specific psychological and physiological responses that occur when smokers are exposed to smoking-related cues.^2,3^ Cigarette craving, as one kind of psychological response, is quickly and strongly triggered on exposure to smoking-related cues.^4^ The intensity of this cue-induced craving is thought to be strongly associated with the likelihood of relapse.^5^ Improving our understanding of cue-induced craving has potential clinical benefits for nicotine addiction interventions.^6^

Previous electroencephalogram (EEG) studies on smoking cue reactivity have demonstrated that cue-induced craving is related to posterior alpha power,^7,8^ P300 event-related potential,^9^ slow positive waves^10^ and low-theta coherence,^11^ suggesting that its neural correlates can range from the regional activity level to the brain network level. However, these traditional EEG analyses mainly focus on the regional activity level, with the brain network level being relatively rarely studied. As the brain network level may provide richer and more complete insights into cue-induced craving,^12^ the current study will explore neural correlates at the brain network level of cue-induced craving in nicotine addiction, reflected by EEG microstates.

EEG microstate analysis is a powerful, inexpensive and clinically translatable neurophysiological method to assess the function of brain networks.^13–15^ It has been shown that EEG topography remains stable for a short period (80–120 ms), then rapidly transitions to another topography and remains stable again.^13,14^ These transient stable EEG topographies are called EEG microstates, which are thought to reflect basic steps in brain information processing.^16^ Abnormal EEG microstates in psychiatric patients have been revealed in the resting state and in response to stimuli, suggesting that EEG microstates could reflect abnormal brain network activity that underlies the pathogenesis of psychiatric disorders such as schizophrenia, panic disorder, depressive disorder and addiction.^17–21^ In particular, in the resting state, smokers were found to show abnormal EEG microstates compared with healthy controls.^21^ However, resting-state studies on nicotine addiction provide a limited understanding of cue-induced craving, which is strongly associated with relapse.^5^ Therefore, it is worth exploring whether the abnormal brain network activity in smokers on exposure to smoking-related cues would be captured by EEG microstates. In the current study, the guided imagery cue reactivity task was used to induce cigarette craving.^6^ Smoking-related imagery scripts were derived from personalised smoking-related experiences of the participants. As the scripts describe smoking-related scenes as well as actions and feelings personalised to the experience of the individual, these smoking-related cues are more lifelike and motivationally powerful for participants compared with image, video and *in vivo* cues.^22^

Previous EEG microstate studies have identified four standard microstate topographies (labelled classes A, B, C and D), which are associated with the auditory network, visual network, salience and memory networks, and dorsal attention network respectively.^13–15^ Interestingly, in previous functional magnetic resonance imaging (fMRI) studies, brain networks found to be activated when substance-dependent individuals were exposed to drug-related cues compared with neutral cues were the visual network, salience network, memory network and dorsal attention network.^23–26^ We thus hypothesised that compared with neutral cues, smoking-related cues could significantly increase one or more parameters (duration, occurrence and contribution) of microstate classes B, C and D. Furthermore, considering the close association between particular brain networks, namely the memory network, salience network and dorsal attention network, and drug craving,^12^ we hypothesised that changes in parameters for classes C and D (smoking condition minus neutral condition) might be associated with changes in cigarette craving. Finally, we also examined the relationship between the changes in parameters of the microstates and nicotine addiction characteristics (e.g. nicotine dependence, number of cigarettes smoked per day and number of years of smoking).

## Method

### Participants

Through online advertisements and fliers, we recruited a total of 40 male smokers. Nicotine dependence was assessed by DSM-5 and the Fagerström Test for Nicotine Dependence (FTND). Other inclusion criteria were as follows: age between 18–50 years; smoking at least 10 cigarettes per day for 2 years or more; and right-handedness. The exclusion criteria were as follows: discomfort with the EEG experimental environment; receipt of any smoking cessation treatment in the previous 3 months. Owing to the low percentage of female smokers in China (2.1%), only male smokers were recruited. For more details about the recruitment, see the supplementary materials available at https://dx.doi.org/10.1192/bjo.2022.641.

The authors assert that all procedures contributing to this work comply with the ethical standards of the relevant national and institutional committees on human experimentation and with the Helsinki Declaration of 1975, as revised in 2008. All procedures involving human participants/patients were approved by the Medical Research Ethics Committee of the First Affiliated Hospital of the University of Science and Technology of China (2022KY-123). All participants signed an informed consent form.

### Guided imagery script generation

Personalised scripts describing experiences of cigarette use and neutral relaxation were generated using the Scene Construction Questionnaire (SCQ) during the interview session.^6^ For smoking-related scripts, participants described a recent situation when they most wanted to smoke and finally smoked a cigarette (e.g. seeing someone else smoking). All situations that contained elements of negative emotions and stress were excluded (e.g. a cigarette after a quarrel). For neutral-related scripts, participants described a recent situation that put them in a calm and peaceful state (e.g. reading a book). All situations that involved cigarettes, friends associated with smoking behaviour or those involving high arousal were excluded. As a manipulation check for situation content, each situation was evaluated twice, first by the researcher and second by two objective independent raters.^6^ For more details, see the supplementary materials. After participants had described each situation and the researcher had checked the content of the situation, participants were given a list of phrases that describe physiological/bodily sensations and were told to circle all of the physiological/bodily sensations that they would experience in the situation in question.^6^ For each participant, a total of four smoking-related and four neutral-related descriptions were completed. The descriptions were edited into the final scripts and audio-taped to a standard length of 60 s. Although these scripts contained individual elements and were therefore personalised, they were of uniform format.^6^

### Imagery training

All participants underwent imagery training, which has been found to effectively improve imagery performance.^6^ Participants were asked to practise imagining five training scripts, which were derived from the Imagery Script Development Procedures Manual and were audio-taped to a standard length of 60 s. At the end of each training trial, participants were asked to rate vividness on a 0–10 visual analogue scale. The result is shown in Supplementary Fig. 1. The baseline imagery ability of the participants was also assessed (see the supplementary materials).

### Experimental procedure

**Fig. 1:**
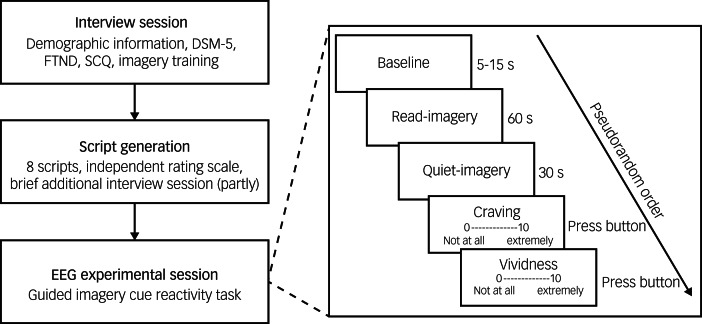
Experimental procedure in our study. FTND, Fagerström Test for Nicotine Dependence; SCQ, Scene Construction Questionnaire; EEG, electroencephalogram. During the read-imagery period, participants were asked to imagine themselves in the situation being described; during the quiet-imagery period, participants continued to imagine until they were told to stop.

The experimental procedure is summarised in Fig. 1. During the interview session, participants provided basic demographic information, including age, years of education, number of cigarettes smoked per day and years of smoking, completed the questionnaires (DSM-5 nicotine dependence, FTND and SCQ) and underwent imagery training. During the EEG experimental session, all participants completed the guided imagery cue reactivity task. We used a block design in the EEG study. There were two blocks (smoking and neutral block) and the four trials in each block were presented in pseudorandom order. Each trial contained a baseline period (5–15 s), a read-imagery period (60 s) and a quiet-imagery period (30 s). During the baseline period, participants closed their eyes and waited for the audio-recording. During the read-imagery period, participants were asked to imagine themselves in the situation being described. During the quiet-imagery period, participants continued to imagine until they were told to stop. Participants then opened their eyes and completed ratings for cigarette craving and vividness on a 0–10 visual analogue scale. To avoid carryover effects, that is, interference caused by smoking-related cues, the smoking block was always presented after the neutral block. Before the EEG experimental session participants received a telephone call instructing them not to smoke for at least 2 h before the session began. Details on ensuring and validating smoking deprivation before the EEG experimental session are given in the supplementary materials.

### EEG acquisition

The EEG data were recorded using a SynAmps RT amplifier (NeuroScan, Charlotte, NC, U.S.). According to the international 10–20 system, 64 electrodes were placed on the participant's scalp. Electrooculogram (EOG) signals and mastoid signals were recorded using four electrodes (HEOL, HEOR, VEOL and VEOU) and two electrodes (M_1_ and M_2_) respectively. The tip of the participant's nose was used as the location for the reference electrode. To prevent interference from electromagnetic noise, AFz was selected as the ground electrode. The impedance of all electrodes was kept under 5 kΩ. The raw EEG signals were digitised at a sampling rate of 1000 Hz.

### EEG data preprocessing

Preprocessing of the raw EEG data was conducted using EEGLAB toolbox (version 14_1_1b) in MATLAB 2020a for Windows (Mathwork Inc., Natick, USA). The raw EEG data were bandpass filtered between 0.1 and 80 Hz, epoched from −1 to 90 s relative to the beginning of the cue onset, and baseline corrected using the interval from −1 to 0 s; 1 to 89 s of each epoch was selected and a conventional recursive least squares algorithm was used to correct for blink artefacts.^27^ Then, the EEG data were segmented into 1 s epochs, and epochs containing amplitude changes exceeding ±100 mV were rejected. For the EEG microstate analysis, data were downsampled at 250 Hz, bandpass filtered between 2 and 20 Hz and re-referenced to the average reference.

### EEG microstate analysis

First, we calculated the global field power (GFP), which represents the standard deviation of the potentials of all electrodes in the topographical map at each time point, by using the following equation:
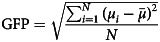


where *N* is the number of electrodes, *μ*_*i*_ is the potential of electrode *i* at a given time point and 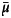 is the mean of the potentials of all electrodes. In the GFP time series, troughs represent a transition from one type of topographical map to another.^14^ The topographical maps at the GFP peaks had the highest signal-to-noise ratio and were selected as the initial prototype maps (Supplementary Fig. 2(a)).^28^

Second, at the individual level, initial prototype maps were submitted to *k*-means clustering, with *k* ranging from 2 to 8. The Krzanowski–Lai (KL) criterion was used to determine the optimal number of microstate classes for further analysis. The optimal *k* was chosen as the *k* corresponding to the second KL maximum value.^14^ Consistent with previous studies, the optimal number of microstate classes was four in our study. We thus obtained individual topographical maps for each participant in the two conditions. For each condition, the individual topographical maps were then averaged across participants using a permutation algorithm.^28^ We thus obtained two sets of topographical maps at the condition level (Supplementary Fig. 2(b)). After that, the overall topographical maps were calculated by averaging the condition-level maps.

Third, the overall topographical maps were fit back to the original GFP peaks for each participant. Based on the maximal spatial correlation between topographies, each GFP peak was identified as one of four classes (Supplementary Fig. 2(c)). Microstate parameters (duration, occurrence and contribution) were then calculated for each class. Specifically, duration represents the average length of time for each class, occurrence represents the total number of each class in 1 s and contribution represents the percentage of the time covered by each class.^14^

### Statistical analyses

Paired *t*-testing was conducted to compare the differences between conditions for craving and vividness ratings. Repeated measures analysis of variance (rm-ANOVA) was performed with the factors ‘condition’ (smoking versus neutral) and ‘microstate class’ (classes A, B, C and D) for duration, occurrence and contribution. Post hoc paired *t*-testing was performed for each class only when the interaction effect was significant. To prevent type I error caused by the multiple-comparison problem, the false discovery rate (FDR) was used to correct significance. Changes in behavioural (e.g. craving ratings) and EEG data (e.g. microstate parameters) were measured by absolute changes (smoking minus neutral) only when significant differences were observed between conditions. All correlation analyses were measured using Pearson's correlation coefficient. The mediation analysis was performed using the bootstrapped method in the PROCESS macro for SPSS for Windows.^29^ Two-tailed *P*-values <0.05 were considered significant.

## Results

### Demographic and clinical characteristics

Table 1 gives demographic and clinical characteristics for the participants.

**Table 1:** Demographic and clinical characteristics of the study population (*n* = 40)

	Mean	s.d.
Age, years	25.9	5.8
Years of education	15.3	2.5
Cigarettes smoked per day, *n*	15.4	5.9
Years of smoking	7.5	5.6
FTND score	4.3	1.6
DSM-5 nicotine dependence symptom count	5.0	1.0

FTND, Fagerström Test for Nicotine Dependence.

### Craving and vividness ratings between conditions

**Fig. 2:**
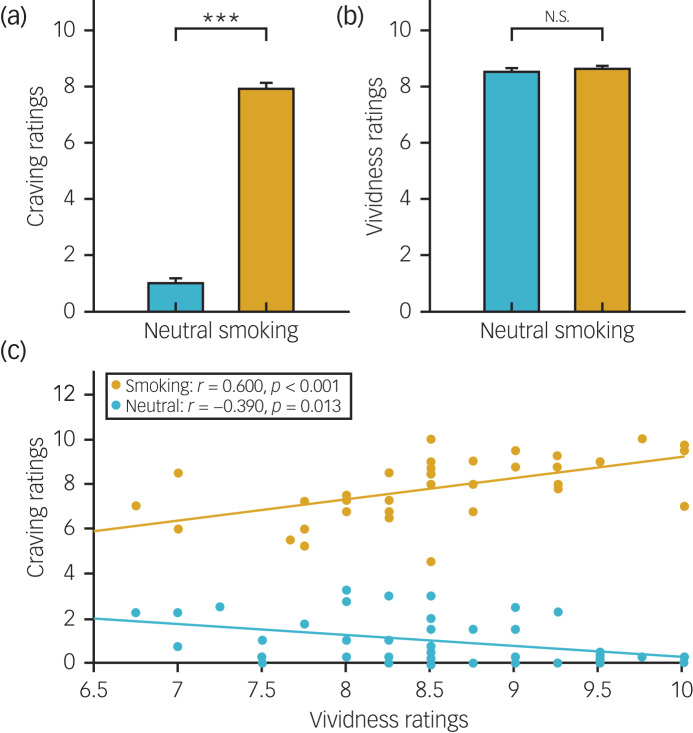
Subjective ratings between conditions. (a) Craving ratings. (b) Vividness ratings. (c) The correlation between craving and vividness ratings. ***P < 0.001; N.S., not significant; error bar, standard error (s.e.).

We found that the craving ratings were significantly higher in the smoking condition than in the neutral condition (*t* = −23.673, *P* < 0.001; Fig. 2(a)). However, no significant differences in vividness ratings were found between conditions (*t* = −0.803, *P* = 0.427; Fig. 2(b)). In addition, we found a significant positive correlation between craving ratings and vividness ratings in the smoking condition (*r* = 0.600, *P* < 0.001; Fig. 2(c)), whereas the opposite correlation was found in the neutral condition (*r* = −0.390, *P* = 0.013; Fig. 2(c)). These results suggest that the guided imagery cue reactivity task is effective in inducing cigarette craving.

### EEG microstate parameters between conditions

**Fig. 3:**
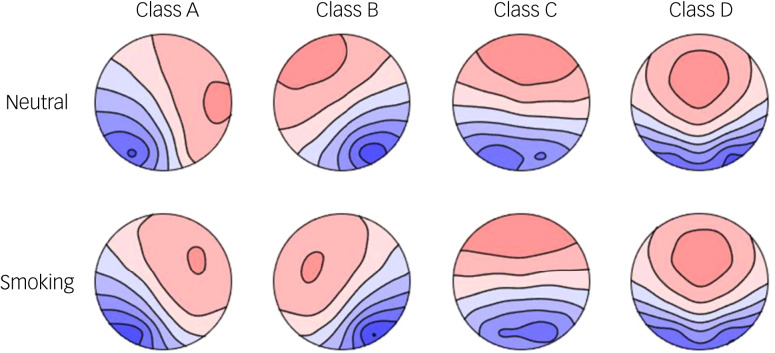
Four microstate topographies (classes A–D) in the neutral condition (top) and smoking condition (bottom).

Figure 3 shows the four microstate topographies in the two conditions, which are very similar to the four standard topographies in previous studies.^30^ The mean global explained variance (GEV) of the four classes was 0.824 (s.d. = 0.043) in the neutral condition and 0.833 (s.d. = 0.044) in the smoking condition. Table 2 shows the comparison of the microstate parameters between conditions.

**Table 2:** Post-hoc paired *t*-test results for the microstate parameters ‘duration’, ‘occurrence’ and ‘contribution’ between conditions^a^

	Smoking condition		Neutral condition		*t*	*P*
	Mean	s.d.	Mean	s.d.		
Duration						
Class A	68.557	8.299	68.160	7.922	−0.653	0.518
Class B	72.945	10.850	71.019	10.771	−3.106	**0.004**
Class C	77.508	23.747	75.435	23.868	−2.850	**0.007**
Class D	86.497	26.291	81.479	23.106	−3.183	**0.003**
Occurrence						
Class A	2.738	0.902	2.973	0.903	6.069	**<0.001**
Class B	3.208	0.796	3.293	0.783	2.200	**0.034**
Class C	3.440	0.685	3.562	0.686	3.303	**0.002**
Class D	3.566	0.579	3.609	0.644	1.408	0.167
Contribution						
Class A	0.189	0.070	0.204	0.069	4.354	**<0.001**
Class B	0.235	0.073	0.233	0.066	−0.409	0.685
Class C	0.267	0.093	0.268	0.091	0.258	0.798
Class D	0.309	0.102	0.295	0.098	−2.495	**0.017**

a. Significant results after false discovery rate correction are shown in bold.

**Fig. 4:**
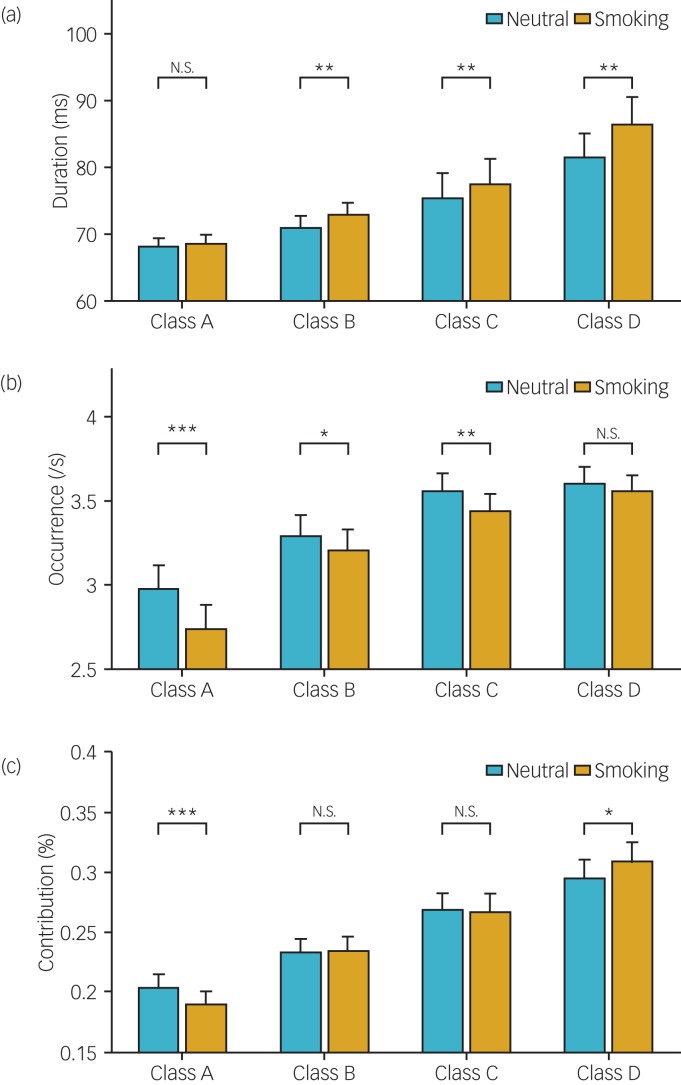
Comparison of the microstate parameters between conditions. (a) Duration. (b) Occurrence. (c) Contribution. ***P < 0.001; **P < 0.01; *P < 0.05; N.S., not significant; error bar, SE.

For duration, the rm-ANOVA showed a significant main effect of condition (*F* = 26.978, *P* < 0.001), a significant main effect of microstate class (*F* = 6.595, *P* < 0.001) and a significant condition × microstate class interaction effect (*F* = 3.798, *P* = 0.012). Post-hoc analysis found that classes B, C and D had longer mean durations in the smoking condition than in the neutral condition (Fig. 4(a) and Table 2).

For occurrence, the rm-ANOVA showed a significant main effect of condition (*F* = 28.721, *P* < 0.001), a significant main effect of microstate class (*F* = 10.646, *P* < 0.001) and a significant condition × microstate class interaction effect (*F* = 6.172, *P* < 0.001). Post-hoc analysis found that the mean occurrence of classes A, B and C was lower in the smoking condition than in the neutral condition (Fig. 4(b) and Table 2).

For contribution, the rm-ANOVA showed a significant condition × microstate class interaction effect (*F* = 5.592, *P* = 0.001) and a significant main effect of microstate class (*F* = 8.960, *P* < 0.001) but no significant main effect of condition (*F* = 0.000, *P* = 1.000). Post-hoc analysis found that the mean contribution of class A was lower and the mean contribution of class D was higher in the smoking condition than in the neutral condition (Fig. 4(c) and Table 2).

### Correlation between EEG microstate parameters and craving ratings

**Fig. 5:**
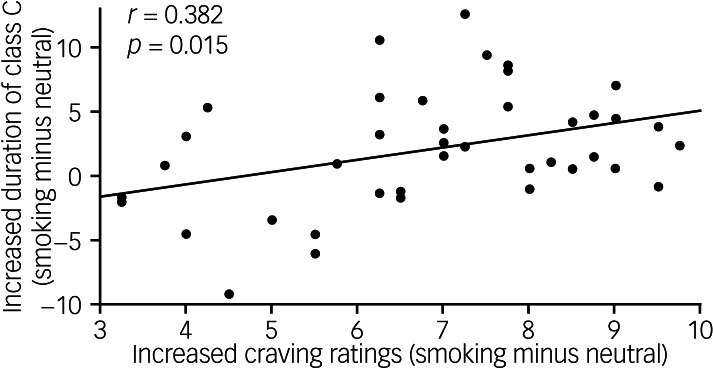
Correlation between increased craving ratings (smoking condition minus neutral condition) and increased duration of microstate class C.

We examined the relationship between the changes in craving ratings and the changes in parameters of microstate classes C and D, which showed significant increases in the smoking condition compared with the neutral condition. Correlation analysis showed a significant positive correlation between the increased craving ratings and the increased duration of class C (*r* = 0.382, *P* = 0.015; Fig. 5). No significant correlation was found between the increased craving ratings and the increased duration and contribution of class D (Supplementary Table 2).

### Correlation between EEG microstate parameters and nicotine addiction characteristics

**Fig. 6:**
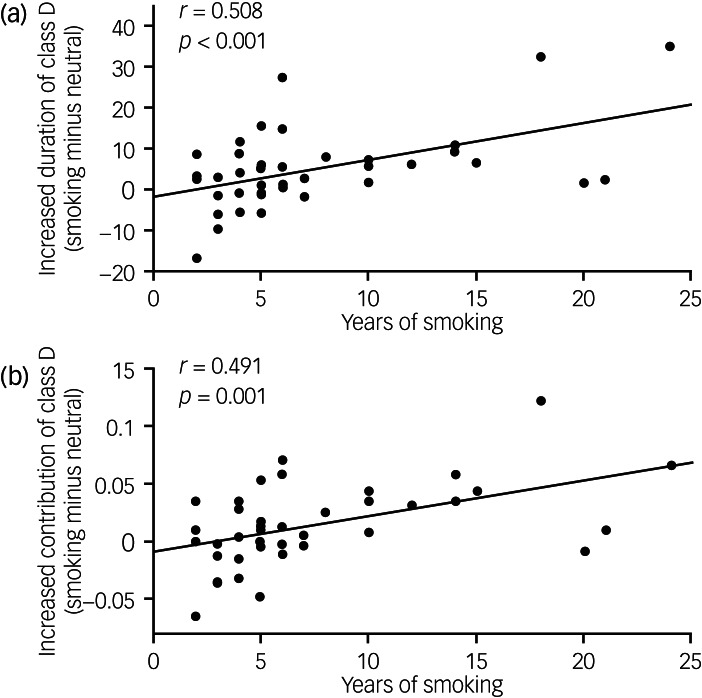
Correlation between years of smoking and (a) the increased duration of microstate class D (smoking condition minus neutral condition) and (b) the increased contribution of microstate class D.

Correlation analysis was performed to explore whether there existed correlation between the nicotine addiction characteristics (FTND, DSM-5, number of cigarettes smoked per day and years of smoking) and the changes in microstate parameters which showed significant differences between conditions. We only found that years of smoking was positively correlated with the increased duration of class D (*r* = 0.508, *P* < 0.001; Fig. 6(a)) and the increased contribution of class D (*r* = 0.491, *P* = 0.001; Fig. 6(b)). The full results are displayed in Supplementary Table 3.

### Relationship between duration of microstate class C and craving ratings

**Fig. 7:**
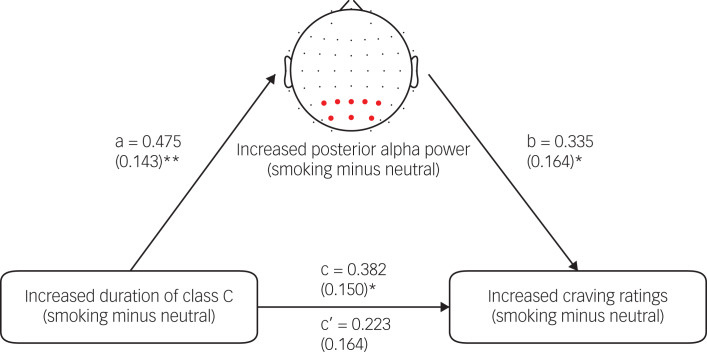
The posterior alpha power mediation effect. The electrodes marked with red dots were used to compute the alpha (8–12 Hz) power. Values above the arrows indicate standardised regression coefficients and values in parentheses indicate the standard error. **P < 0.01; *P < 0.05.

As noted above, our results suggested that the increased duration of microstate class C was significantly correlated with the increased craving ratings. Given the experimental paradigm similarities between our study and the study that found class C to be associated with the memory network,^15^ the finding might suggest the important role of smoking-related memories in inducing cigarette craving. Here, mediation analysis was performed to further support this speculation. A previous study found a significant positive association between the duration of class C and posterior alpha power.^31^ Importantly, posterior alpha power was thought to be associated with memory processes.^31^ We thus assumed that the association between increased duration of class C and increased craving ratings might be explained by increased posterior alpha power. First, we found that there was a significant increase in posterior alpha power in the smoking condition compared with the neutral condition (*t* = −3.529, *P* = 0.001). Second, correlation analysis showed that there was a significant positive correlation between increased duration of class C and increased posterior alpha power (*r* = 0.475, *P* = 0.002), supporting the finding from the previous study.^31^ Finally, mediation analysis showed that increased posterior alpha power fully mediated the relationship between the increased duration of class C and the increased craving ratings (Fig. 7). For more details about the mediation analysis, see the Supplementary materials.

## Discussion

In this study, we examined the effects of exposure to smoking-related cues (conditions) on EEG microstates in smokers. First, we found that the smoking condition elicited a significant increase in microstate classes B (duration), C (duration) and D (duration and contribution) compared with the neutral condition. Second, we found a significant positive correlation between increased duration of class C and increased craving ratings, which was fully mediated by increased posterior alpha power. Finally, we also found that the increased duration and contribution of class D were both positively correlated with years of smoking.

Our study revealed a significant increase in one or more parameters of microstate classes B, C and D in the smoking condition, supporting our first hypothesis. Previous fMRI studies have shown that drug-related cues could elicit significant activation of the visual network, salience network, memory network and dorsal attention network in substance-dependent individuals.^23–26^ This is consistent with findings from previous EEG microstate studies,^13–15^ suggesting an association between class B and the visual network, class C and both the salience network and the memory network, and class D and dorsal attention network. In addition, we found a significant decrease in one or more parameters of class A in the smoking condition. Since class A has been shown to be associated with the auditory network,^13–15^ it is prudent to speculate that imagining the neutral situation is more dependent on the guidance of the audio-scripts than imagining the smoking situation. Similar to our results, a previous study reported abnormal EEG microstates induced by methamphetamine-related cues in methamphetamine-dependent individuals.^20^ Taken together, our results suggest that the abnormal brain network activity in smokers on exposure to smoking-related cues would be captured by EEG microstates.

We found that abnormal changes in the duration of microstate class C were associated with changes in cigarette craving, that is, the participants who had a greater increase in the duration of class C also had a greater increase in craving rating in the smoking condition compared with the neutral condition. This result supports our second hypothesis. Given the experimental paradigm similarities between the present study and the study that found class C to be associated with the memory network,^15^ our result might, to some extent, suggest the important role of smoking-related memories in inducing cigarette craving on exposure to smoking-related cues. This speculation is supported by the result showing the mediating effect of posterior alpha power. Posterior alpha power has been shown to be associated with memory processes.^31^ Previous studies also revealed the relationship between cigarette craving and cue-elicited activation of the memory network.^32,33^ Our result suggests that the duration of class C is a novel biomarker for cue-induced craving that could be targeted for the treatment and assessment of nicotine addiction interventions.

We also found that abnormal increases in the duration and contribution of microstate class D were both associated with years of smoking. Owing to the relationship between class D and the dorsal attention network,^13–15^ our result might indicate that smokers who have a more years of smoking would recruit more cognitive resources on exposure to smoking-related cues. Similar to our findings, a previous study reported that nicotine dependence severity was associated with cue-elicited activation of the attention network.^33^ Our result suggests that the duration and contribution of class D could reflect the relationship between years of smoking and cue-elicited activation of the dorsal attention network.

In recent years, EEG-based neurofeedback has increasingly been used in the treatment of addiction.^34,35^ Compared with traditional neurofeedback, microstate neurofeedback might have unique advantages. The relationship between microstates and brain networks makes it easier to modulate the target brain network in real time through EEG-based neurofeedback. In fact, there has been preliminary research demonstrating the feasibility of microstate neurofeedback: participants successfully modulated the contribution of microstate class D, which was found to be shorter in schizophrenia.^36^ Inspired by a novel neurofeedback training approach developed by Bu et al that modulated abnormal EEG features (e.g. P300) induced by smoking-related cues,^34^ we suggest that abnormal EEG microstates in smoking cue reactivity could also be targeted in neurofeedback modulation . In addition, the duration of class C might be a valid physiological indicator for assessing the efficacy of nicotine addiction interventions.

### Limitations and future research

Several limitations of this study must be noted. First, only male smokers were recruited, and we limited the age and years of education in the inclusion criteria, so whether our findings could be generalised to the entire smoking population remains unclear. In future, we will consider exploring abnormal EEG microstates induced by smoking-related cues in different gender, age and educational level groups. Second, our study lacked a healthy control group, primarily because the smoking-related imagery scripts were derived from participants’ personalised smoking-related experiences, which are lacking in healthy controls.^6^ Therefore, we can only tentatively conclude that our findings might be specific to nicotine addiction; more studies and evidence are needed to support our findings. Although numerous studies have suggested that cue reactivity is unique to substance-dependent individuals,^37,38^ we recommend that recreational smokers should be recruited as healthy controls in future studies, thus further exploring whether non-dependent individuals would show abnormal EEG microstates on exposure to smoking-related cues.

## Data Availability

The data that support the findings of this study are available from the corresponding author on reasonable request.

## Supplementary material

For supplementary material accompanying this paper visit http://doi.org/10.1192/bjo.2022.641. click here to view supplementary material
