# Supplementary material for: Correlation between abnormal brain network activity and electroencephalogram microstates on exposure to smoking-related cues
Source: BJPsych Open. 2023 Jan 31;9(2):e31. doi: 10.1192/bjo.2022.641 (PMC9970173; doi:10.1192/bjo.2022.641)
Supplement: Supplementary file 1 [file S205647242200641Xsup001.docx]

**Supplementary materials**

**Abnormal EEG microstates induced by smoking-related cues correlated with cigarette craving and years of smoking in nicotine addiction**

Hefan Gan, Junjie Bu, Ginger Qinghong Zeng, Huixing Gou, Mengyuan Liu, Guanbao Cui and Xiaochu Zhang

**Method**

###### Participants

Information about the inclusion criteria (age, number of cigarettes smoked per day, years of smoking, and right-handedness) and the exclusion criteria (received any smoking cessation treatment in the last 3 months) were acquired by the demographic information questionnaire during the interview session. The inclusion criteria (nicotine dependence) was evaluated by a licensed psychiatrist using *The Diagnostic and Statistical Manual of Mental Disorders, Fifth Edition* (*DSM-V*) and The Fagerström Test for Nicotine Dependence (FTND) during the interview session. Information about the exclusion criteria (discomfort with the EEG experimental environment) was acquired by participants’ self-reports during the EEG experimental session.

###### Guided imagery script generation

As a manipulation check for situation content, each situation was evaluated twice, first by the researcher during the interview session, and second by two objective independent raters after the interview session. To be specific, for neutral situations, each participant was told to describe four neutral situations during the interview session. After the participant completed the description of each neutral situation, the researcher would check and ensure that the situation does not contain obvious stimulating, arousing, negative emotional, and stressful elements, and that the participant does not have any thoughts of smoking in this situation. For situation that involve reading a book or watching a movie, the researcher would also check and ensure that the material being read or watched does not contain stimulating, arousing, negative emotional, and stressful content (e.g. reading a book with exciting fighting content). After the interview session, each neutral situation was evaluated by two objective independent raters using 5-point independent rating scales (with 1 = very slightly or not at all, and 5 = very strongly) for arousing content. Negative emotional and stressful content of neutral situation were also rated as they might be cues to trigger smoking craving. Neutral situation rated as 3 or above for arousing, negative emotional, or stressful content by any of the independent raters was excluded, and the participant was told to describe a new situation in the brief additional interview session. For smoking situations, each participant was told to describe four smoking situations during the interview session. After the participant completed the description of each smoking situation, the researcher would check and ensure that the situation does not contain obvious negative emotional or stressful elements. After the interview session, each smoking situation was evaluated by the two objective independent raters for negative emotional and stressful content. Smoking situation rated as 3 or above for negative emotional or stressful content by any of the independent raters was excluded, and the participant was told to describe a new situation in the brief additional interview session. The details about the number of situations collected and excluded for each participant are shown in supplementary table 1.

**Supplementary table 1** The details about the number of situations collected and excluded for each participant.

| Situation type | n | Number of situations collected after interview session | Number of situations excluded by  independent raters | Total number of situations collected | Exclusion percentage |
| --- | --- | --- | --- | --- | --- |
| Smoking | 1 | 4 | 2 | 6 | 33.3% |
|  | 4 | 4 | 1 | 5 | 20.0% |
|  | 35 | 4 | 0 | 4 | 0.00% |
| Neutral | 3 | 4 | 1 | 5 | 20.0% |
|  | 37 | 4 | 0 | 4 | 0.00% |

Total number of situations collected = number of situations collected after interview session + number of situations collected in the brief additional interview session; exclusion percentage = number of situations excluded by independent raters / total number of situations collected.

###### Imagery training

A linear regression analysis revealed that there was a strong and significant positive correlation between the number of training trials and the mean vividness ratings (*r* = 0.965, *P* = 0.008, Fig. S1), suggesting that participants could actively participate in imagining situations after imagery training. Also, we have collected participants’ baseline imagery ability using Vividness of Visual Imagery Questionnaire (VVIQ). Participants were asked to imagine four suggested scenes and then self-rate the vividness of their visual imagery on select details on a 7-point scale (with 1 = perfectly clear and as vivid as normal vision, and 7 = no image at all). For example, participants were asked to visualize a rising sun and rate their ability to vividly visualize certain details, such as ‘The sky clears and surrounds the sun with blueness’ and ‘A rainbow appears’. The VVIQ has demonstrated an internal consistency of 0.88, as measured by Cronbach’s alpha. Participants reporting average or above levels of imagery ability (no more than 56, total VVIQ score: 112) were considered to have good imagery ability. The mean VVIQ score of the participants was 32.55 (s.d. = 9.22), ranging from 16 to 52. Therefore, no participants were excluded because of low imagery ability.


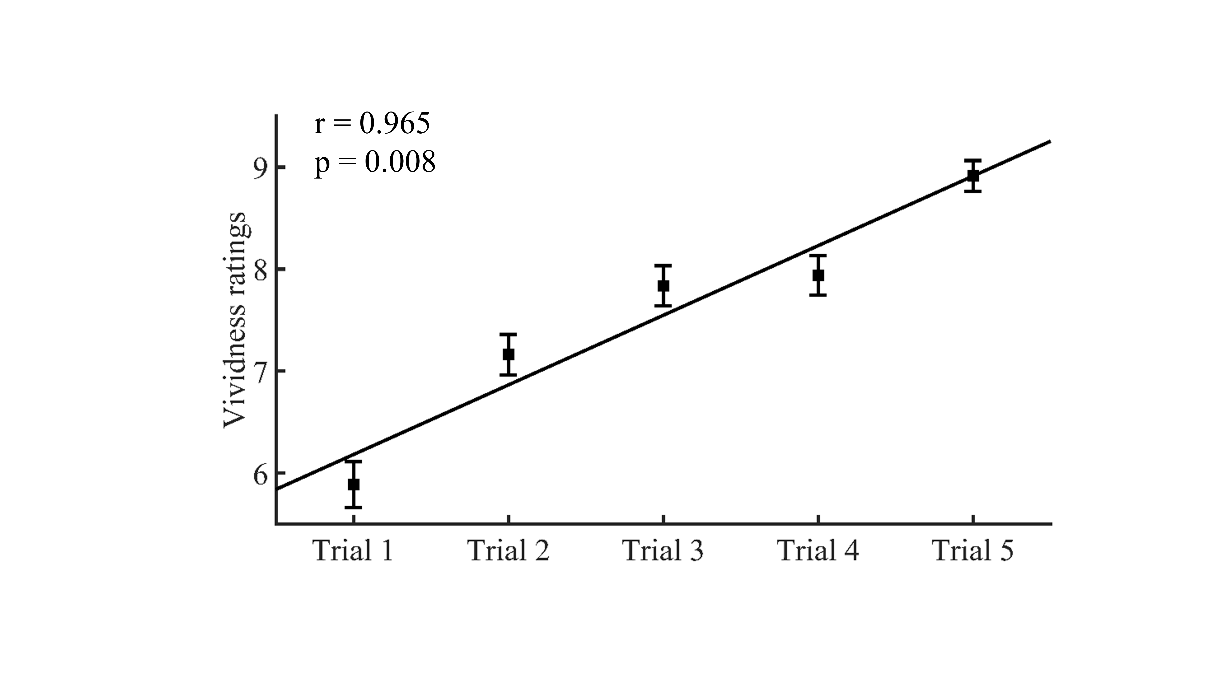


**Fig. S1** The mean rating of vividness increased with the number of training trials. Error bar: standard error (SE).

###### Experimental procedure

The following methods were used to ensure and validate smoker’s 2-hour deprivation before the EEG experimental session. First, prior to the EEG recording, participants were in the smoke-free laboratory under the supervision of the researcher for about two hours (mainly for rest, experimental instruction, questionnaire completion, and EEG preparation). Second, we have examined the exhaled carbon monoxide (CO) concentration (piCO^TM^ Smokerlyzer, Bedfont) to assess smoking status. Participants with the exhaled CO level of 10 ppm or less were considered to be in abstinence state. In our study, the mean CO level of participants was 4.98 ppm (s.d. = 1.54). No participant’s CO level was more than 10 ppm. Third, we collected the time participants smoked their last cigarette before the EEG experimental session. The mean duration of abstinence for participants was 6.34 hours (s.d. = 2.29). No participant’s duration of abstinence was less than 2 hours.

###### EEG microstate analysis

**
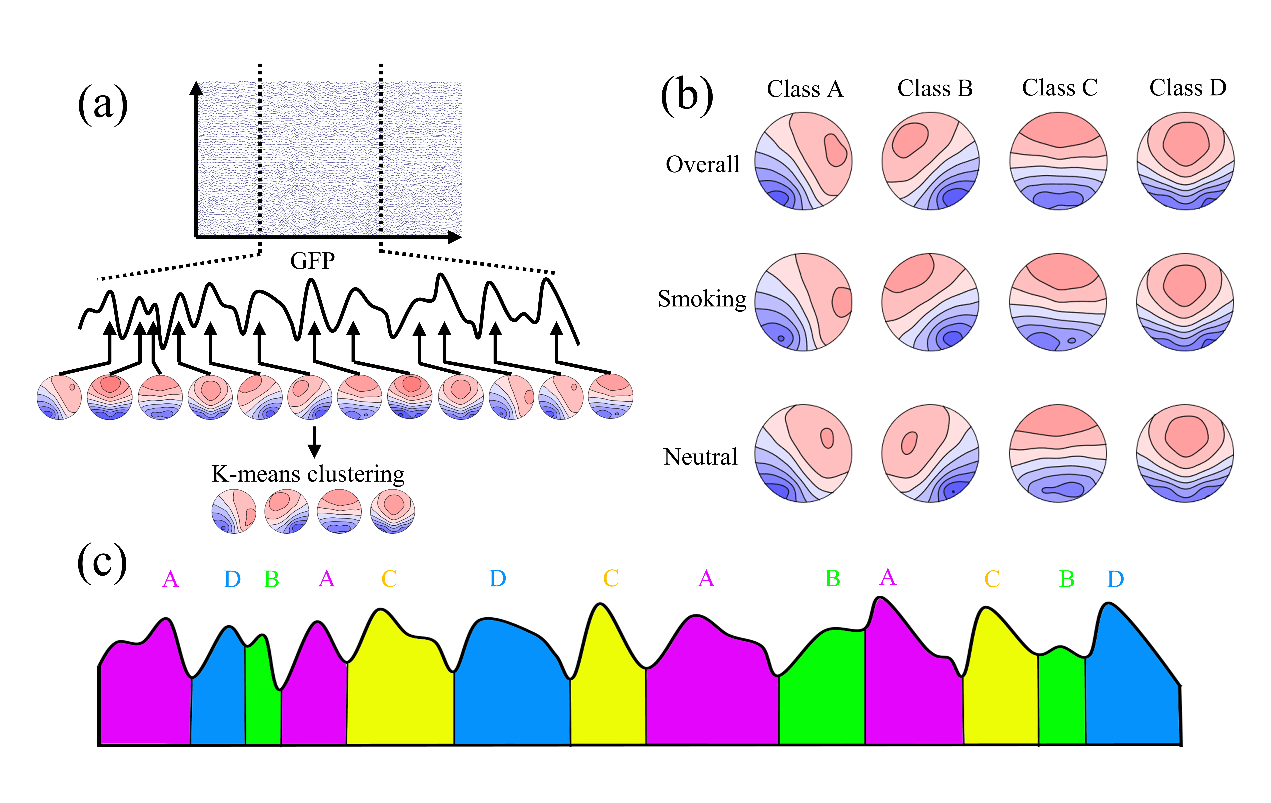
**

**Fig. S2** Overview of the process of EEG microstate analysis.

**Results**

###### Correlation between EEG microstate parameters and craving ratings

**Supplementary Table 2** The correlation between the increased craving ratings and the increased microstate parameters of class C and D (smoking minus neutral). Significant results after FDR correction were marked in bold.

|  | **Increased craving ratings** | |
| --- | --- | --- |
|  | ***r*** | ***P*** |
| **Duration** |  |  |
| **Increased class C** | 0.382 | **0.015** |
| **Increased class D** | 0.038 | 0.815 |
| **Contribution** |  |  |
| **Increased class D** | -0.063 | 0.699 |

###### Correlation between EEG microstate parameters and nicotine addiction characteristics

**Supplementary Table 3** The correlation between the clinical characteristics of nicotine addiction and the changes in EEG microstate parameters (smoking minus neutral). Significant results after FDR correction were marked in bold.

|  | **FTND** | | ***DSM-V*** | | **Cigarette/day** | | **Years of smoking** | |
| --- | --- | --- | --- | --- | --- | --- | --- | --- |
|  | ***r*** | ***P*** | ***r*** | ***P*** | ***r*** | ***P*** | ***r*** | ***P*** |
| **Duration** |  |  |  |  |  |  |  |  |
| **Increased class B** | -0.099 | 0.545 | -0.072 | 0.658 | 0.157 | 0.334 | -0.106 | 0.514 |
| **Increased class C** | -0.090 | 0.581 | 0.046 | 0.777 | -0.175 | 0.281 | -0.323 | 0.042 |
| **Increased class D** | 0.132 | 0.417 | 0.008 | 0.963 | 0.210 | 0.194 | 0.508 | **<0.001** |
| **Occurrence** |  |  |  |  |  |  |  |  |
| **Decreased class A** | 0.084 | 0.607 | 0.111 | 0.497 | -0.059 | 0.716 | 0.011 | 0.948 |
| **Decreased class B** | -0.151 | 0.353 | -0.184 | 0.255 | -0.137 | 0.400 | -0.068 | 0.676 |
| **Decreased class C** | -0.026 | 0.874 | 0.059 | 0.720 | -0.154 | 0.342 | -0.300 | 0.060 |
| **Contribution** |  |  |  |  |  |  |  |  |
| **Decreased class A** | 0.100 | 0.541 | 0.167 | 0.304 | -0.028 | 0.862 | -0.152 | 0.349 |
| **Increased class D** | 0.127 | 0.434 | 0.012 | 0.944 | 0.216 | 0.181 | 0.491 | **0.001** |

FTND: Fagerström Test for Nicotine Dependence; *DSM-V: Diagnostic and Statistical Manual of Mental Disorders, Fifth Edition.*

###### The relationship between the increased duration of class C and the increased craving ratings was fully mediated by the increased posterior alpha power

In the first step of the mediation analysis, the increased posterior alpha power was regressed on the increased duration of class C. Higher increased duration of class C significantly predicted higher increased posterior alpha power (β = 0.475, SE = 0.143, *t* = 3.323, *P* = 0.002). Secondly, the increased craving ratings was regressed on the increased duration of class C. Higher increased duration of class C significantly predicted higher increased craving ratings (β = 0.382, SE = 0.150, *t* = 2.544, *P* = 0.015). Thirdly, the increased craving ratings was regressed on the increased duration of class C together with the increased posterior alpha power. Higher increased posterior alpha power significantly predicted higher increased craving ratings (β = 0.335, SE = 0.164, *t* = 2.045, *P* = 0.048). The relationship between the increased duration of class C and the increased craving ratings decreased in strength with the presence of the increased posterior alpha power and was not significant (β = 0.223, SE = 0.164, *t* = 1.361, *P* = 0.182). The mediation analysis indicated that the increased posterior alpha power fully mediated the effect of the increased duration of class C on the increased craving ratings.
